# The diagnostic and prognostic role of novel biomarkers in anti-neutrophil cytoplasmic antibody-associated vasculitis

**DOI:** 10.3389/fimmu.2025.1588287

**Published:** 2025-06-17

**Authors:** Ruohan Yu, Lina Zhang, Jing Zhang, Ting Long, Ji Li, Yadan Zou, Shangxi Wang, Shengguang Li

**Affiliations:** ^1^ Department of Rheumatology and Immunology, Peking University International Hospital, Beijing, China; ^2^ Health Management Center, Peking University International Hospital, Beijing, China

**Keywords:** monocyte-to-lymphocyte ratio, systemic immune-inflammation index, systemic inflammation response index, disease activity, prognosis

## Abstract

**Background:**

ANCA-associated vasculitis (AAV) is a group of autoimmune diseases characterized by small vessel inflammation, diagnosed primarily through clinical features, histopathology, and ANCA testing. Novel biomarkers derived from routine blood counts, such as neutrophil-to-lymphocyte ratio (NLR), platelet-to-lymphocyte ratio (PLR), monocyte-to-lymphocyte ratio (MLR), systemic immune-inflammation index (SII), and systemic inflammation response index (SIRI), may support disease assessment. This study evaluated their utility in distinguishing AAV patients, reflecting disease activity, and predicting prognosis.

**Methods:**

In this retrospective case-control study, 65 AAV patients and 65 age- and sex-matched healthy controls were enrolled. AAV diagnosis adhered to the 2012 Chapel Hill Consensus and the American College of Rheumatology 1990 criteria. NLR, PLR, MLR, SII, and SIRI were calculated from complete blood counts. Disease activity (Birmingham Vasculitis Activity Score, BVAS), extent (Disease Extent Index, DEI), damage (Vasculitis Damage Index, VDI), and prognosis (Five-Factor Score, FFS 2009) were assessed. Statistical analyses included Mann-Whitney U tests, Spearman correlations, and receiver operating characteristic (ROC) curves to evaluate discriminatory and predictive capacities.

**Results:**

AAV patients exhibited significantly higher NLR (6.94 ± 0.76 vs. 1.88 ± 0.08), PLR (242.44 ± 23.09 vs. 125.97 ± 4.34), MLR (0.44 ± 0.03 vs. 0.20 ± 0.01), SII (1813.71 ± 221.85 vs. 446.62 ± 22.40), and SIRI (3.19 ± 0.31 vs. 0.72 ± 0.06) compared to controls (all P < 0.001). ROC analysis showed strong discriminatory power, with SIRI (AUC = 0.902) and NLR (AUC = 0.885) performing best. NLR, PLR, SII, and SIRI correlated positively with BVAS (rs = 0.325-0.356, *P* < 0.01) and FFS 2009 (rs = 0.358-0.386, *P* < 0.05), and all markers correlated with DEI (rs = 0.396-0.488, *P* < 0.01), but not VDI. For predicting active disease (BVAS ≥ 15), SII had the highest AUC (0.726, *P* = 0.003).

**Conclusions:**

NLR, PLR, MLR, SII, and SIRI effectively distinguish AAV patients from controls and reflect disease activity, extent, and prognosis. While not standalone diagnostic tools, these markers offer valuable support to standard AAV assessment, particularly in challenging cases. Their accessibility suggests potential for enhancing clinical management, pending validation in larger cohorts.

## Introduction

Antineutrophil cytoplasmic antibody (ANCA)-associated vasculitis (AAV) comprises a group of rare autoimmune diseases characterized by necrotizing inflammation of small blood vessels, including capillaries, venules, and arterioles (1). Clinically, AAV is classified into three main subtypes: granulomatosis with polyangiitis (GPA), microscopic polyangiitis (MPA), and eosinophilic granulomatosis with polyangiitis (EGPA), each defined by distinct clinical and pathological features (2). The disease often affects multiple organs, such as the kidneys, lungs, skin, and nervous system, and can lead to severe outcomes, including organ failure, if not addressed promptly (3). Consequently, timely recognition and accurate monitoring of disease activity are critical to improving patient outcomes in AAV (4).

The diagnosis of AAV relies on a combination of clinical manifestations, histopathological findings, and ANCA testing, as outlined in established criteria such as the 2012 Chapel Hill Consensus Conference nomenclature and the 2022 ACR/EULAR classification standards (1, 4). However, challenges persist: ANCA testing varies in sensitivity and specificity across subtypes, and approximately 10-20% of patients may present as ANCA-negative, complicating diagnosis (5). Disease activity is typically monitored using tools like the Birmingham Vasculitis Activity Score (BVAS) (6), but the BVAS is intricate and less suitable for outpatient settings due to its complexity. And these methods can sometimes lack precision or fail to capture subtle changes in disease status. These limitations highlight the need for additional tools to support the diagnostic process and enhance disease assessment.

Inflammation is a central driver of AAV pathogenesis, involving dysregulated activation of immune cells such as neutrophils, monocytes, and lymphocytes (7). In recent years, hematological indices derived from routine blood counts, such as the neutrophil-to-lymphocyte ratio (NLR), platelet-to-lymphocyte ratio (PLR), monocyte-to-lymphocyte ratio (MLR), systemic immune-inflammation index (SII), and systemic inflammation response index (SIRI), have emerged as promising indicators of inflammation in various autoimmune and inflammatory conditions (8–10). These markers are appealing due to their simplicity, affordability, and availability, making them practical for widespread clinical use.

Previous studies have reported elevated NLR and PLR in AAV patients, associating them with disease activity and prognosis (11, 12). However, the roles of MLR, SII, and SIRI in AAV remain underexplored, and comprehensive evaluations of multiple markers in a single cohort are limited. Importantly, while these markers cannot independently diagnose or classify AAV, they may serve as supportive tools to complement established diagnostic methods and aid in disease monitoring. This study aims to systematically assess NLR, PLR, MLR, SII, and SIRI in AAV patients, examining their utility in distinguishing AAV from healthy states, reflecting disease activity, and predicting prognosis.

## Materials and methods

### Study design

This retrospective case-control study was conducted to evaluate the utility of NLR, PLR, MLR, SII and SIRI in supporting the assessment and monitoring of AAV. The study compared these markers between AAV patients and healthy controls and examined their associations with disease activity, extent, and prognosis to explore their potential as complementary clinical tools.

### Patients and controls

All hospitalized patients with confirmed diagnosis of ANCA-associated vasculitis in Peking University International hospital from 2015 to 2024 were included for analysis. AAV was diagnosed based on the American College of Rheumatology 1990 criteria and then reclassified by the 2012 revised Chapel Hill Consensus Conferences Nomenclature of Vasculitis (1, 13, 14). Exclusion Criteria: Presence of concurrent autoimmune diseases; Evidence of active infection or malignancy at the time of assessment.

The results of the blood routine test of 65 healthy controls were retrospectively extracted from Health Examination Center. Controls had no history of autoimmune, inflammatory, or infectious diseases. This study was approved by the Ethics Committee of Peking University International Hospital(2024-KY-0046-01), who waived the need for patient written informed consent, as this was a retrospective study.

### Clinical and laboratory data

Clinical and laboratory data were collected from medical records. Laboratory results, including white blood cell (WBC), neutrophil, lymphocyte, monocyte and platelet count, erythrocyte sedimentation rate (ESR), C-reactive protein (CRP), serum creatinine were detected by routine methods. The following index was calculated based on the blood routine examination: NLR = neutrophil count/lymphocyte count; PLR=platelet/lymphocyte count; MLR=Monocyte count/Lymphocyte count; systemic immune-inflammation index (SII)=PLT count × neutrophil count/lymphocyte count; The systemic inflammation response index (SIRI) = (neutrophil count × monocyte count)/lymphocyte count.

### Disease assessment tools

Disease Activity: Assessed using the 2003 Birmingham Vasculitis Activity Score (BVAS) (6), with a score ≥15 indicating active disease. Disease Extent: Measured by the Disease Extent Index (DEI) to evaluate organ involvement. Vasculitis Damage: Quantified with the Vasculitis Damage Index (VDI) for irreversible organ damage (15). Prognosis: Evaluated using the 2009 Five-Factor Score (FFS 2009) to assess mortality risk (16).

### Statistical analysis

SPSS 21 was used for the statistical Analysis. Continuous variable was expressed as mean ± standard deviation (SD), and categorical data are expressed as percentages. To compare the difference between two groups, Mann–Whitney U test was used for continuous variables and chi square test was used for nominal variables. Spearman correlation analysis was performed to assess the correlation between the variables. Receiver operating characteristic (ROC) curve analysis was conducted to determine the sensitivity and specificity of the new inflammatory markers in diagnosing of AAV. The cut-off value was calculated. *P* < 0.05 was considered statistically significant.

## Results

### Baseline characteristics of AAV patients and healthy controls

The study cohort comprised 65 patients with ANCA-associated vasculitis (AAV) and 65 age- and sex-matched healthy controls. The mean age of AAV patients were 65.99 ± 13.55 years, with a mean disease duration of 30.61 ± 69.8 months. Subtype distribution included 36 patients (55.4%) with granulomatosis with polyangiitis (GPA), 24 (36.9%) with microscopic polyangiitis (MPA), and 5 (7.7%) with eosinophilic granulomatosis with polyangiitis (EGPA). The mean value of ESR and CRP was 37.78 ± 36.97mm/h and 38.12 ± 51.24 mg/L, respectively. The mean creatinine value of the patients was 390.38 ± 334.82 μmol/L. No significant differences were observed between AAV patients and controls in age (65.60 ± 0.44 years in controls, *P* = 0.087) or sex distribution (31 males vs. 32 males, *P* = 0.861).

Compared to controls, AAV patients had significantly higher white blood cell (WBC) counts (9.30 ± 3.33 vs. 6.03 ± 0.17 ×10^9^/L, *P* < 0.001), neutrophil counts (7.14 ± 3.23 vs. 3.55 ± 0.13 ×10^9^/L, *P* < 0.001), and monocyte counts (0.53 ± 0.26 vs. 0.39 ± 0.04 ×10^9^/L, *P* < 0.001), alongside lower lymphocyte counts (1.38 ± 0.80 vs. 1.96 ± 0.06 ×10^9^/L, *P* < 0.001) and hemoglobin levels (107.87 ± 24.14 vs. 141.35 ± 1.32 g/L, *P* < 0.001). Platelet counts did not differ significantly (255.02 ± 97.68 vs. 236.23 ± 5.88 ×10^9^/L, *P* = 0.531). The NLR, PLR, MLR, SII, and SIRI were markedly elevated in AAV patients compared to controls (all *P* < 0.001). Detailed baseline data are presented in **Table 1**.

**Table 1:** Characteristics of patients with AAV and healthy controls.

Variables	AAV(N=65)	Healthy controls(N=65)	*P*
sex			0.861
male	31	32	
female	34	33	
Age (year)	65.99 ± 13.55	65.60 ± 0.44	0.087
Disease course (month)	30.61 ± 69.8	–	
diagnosis			
EGPA	5	–	
GPA	36	–	
MPA	24	–	
WBC (×10^9^/L)	9.30 ± 3.33	6.031 ± 0.17	<0.001
Hemoglobin (g/L)	107.87 ± 24.14	141.35 ± 1.32	<0.001
platelet (×10^9^/L)	255.02 ± 97.68	236.23 ± 5.88	0.531
monocyte (×10^9^/L)	0.53 ± 0.26	0.39 ± 0.04	<0.001
neutrophil (×10^9^/L)	7.14 ± 3.23	3.55 ± 0.13	<0.001
lymphocyte (×10^9^/L)	1.38 ± 0.80	1.96 ± 0.06	<0.001
NLR	6.94 ± 0.76	1.88 ± 0.08	<0.001
PLR	242.44 ± 23.09	125.97 ± 4.34	<0.001
MLR	0.44 ± 0.03	0.20 ± 0.01	<0.001
SII	1813.71 ± 221.85	446.62 ± 22.40	<0.001
SIRI	3.19 ± 0.31	0.72 ± 0.06	<0.001

### Ability of inflammatory markers to distinguish AAV patients

**Figure 1:**
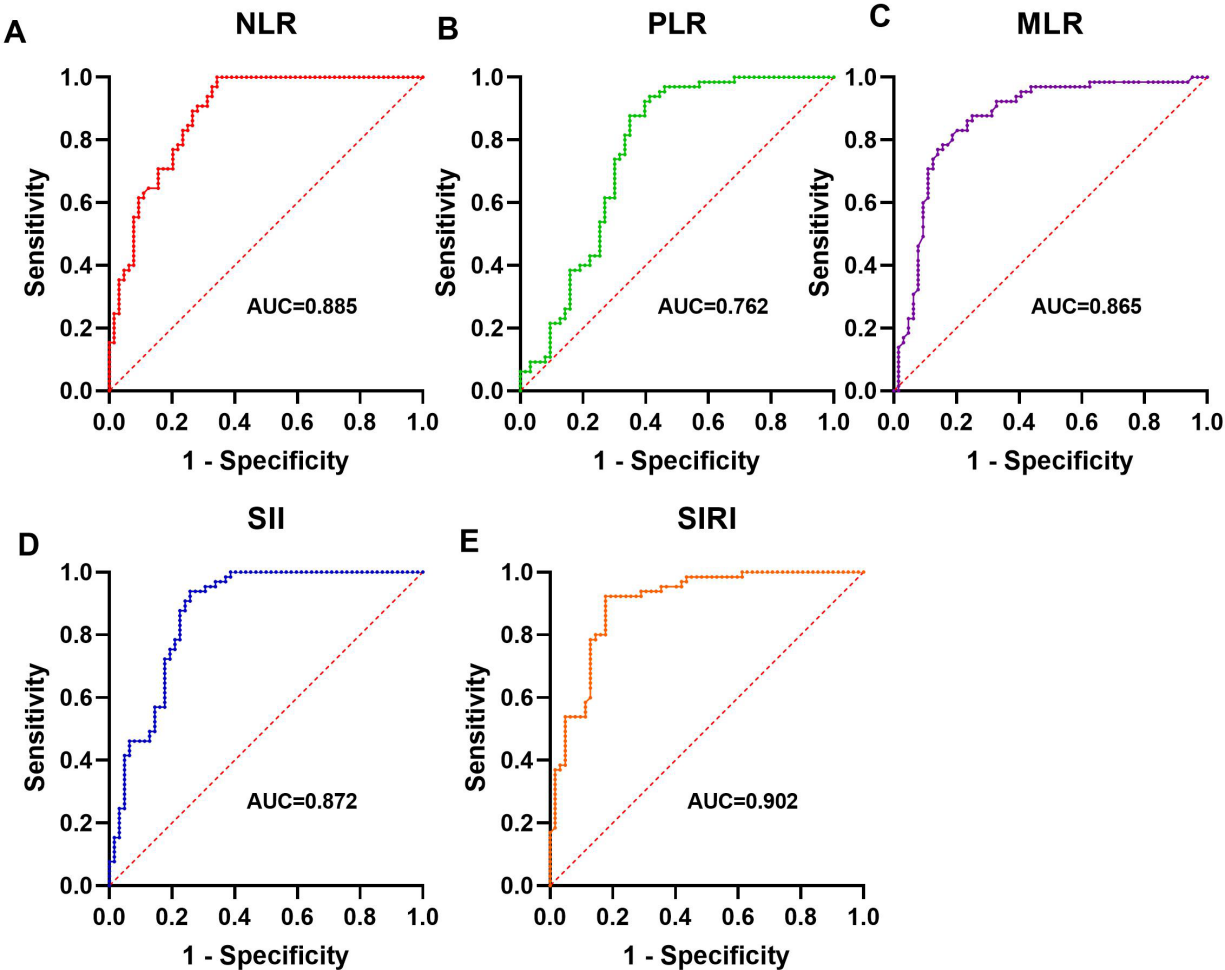
The significance of NLR, PLR, SII and SIRI in diagnosing AAV ROC analysis showing the ability of NLR **(A)**, PLR **(B)**, MLR **(C)**, SII **(D)**, and SIRI **(E)** in differentiating AAV patients from healthy controls.

Receiver operating characteristic (ROC) analysis assessed the capacity of NLR, PLR, MLR, SII, and SIRI to differentiate AAV patients from healthy controls. All markers exhibited significant discriminatory ability (*P* < 0.001). SIRI showed the highest AUC (0.902, 95% CI: 0.848-0.955), followed by NLR (0.885, 95% CI: 0.827-0.972) and SII (0.872, 95% CI: 0.808-0.936). Optimal cutoff values, along with sensitivity and specificity, are summarized in **Table 2**, and visualized in **Figure 1**, indicating their potential to support identification of AAV patients when used alongside standard diagnostic criteria.

**Table 2:** The cut-off value of NLR, PLR, MLR, SII, and SIRI for diagnosing AAV.

Variable	AUC	*P*	95%CI	Sensitivity (%)	Specificity (%)	Cut-off value
NLR	0.885	<0.001	0.827-0.972	0.644	1	3.776
PLR	0.762	<0.001	0.675-0.849	0.644	0.877	159.548
MLR	0.865	<0.001	0.798-0.932	0.814	0.831	0.232
SII	0.872	<0.001	0.808-0.936	0.729	0.938	721.226
SIRI	0.902	<0.001	0.848-0.955	0.847	0.923	1.059

### Associations with disease activity, extent, and prognosis

Spearman correlation analysis explored relationships between inflammatory markers and disease parameters. NLR, PLR, SII, and SIRI were positively correlated with BVAS (rs = 0.347, *P* = 0.005; rs = 0.325, *P* = 0.009; rs = 0.356, *P* = 0.004; rs = 0.344, *P* = 0.006, respectively), while MLR showed no significant association (rs = 0.203, *P* = 0.108). All markers correlated positively with DEI (NLR: rs = 0.396, *P* = 0.009; PLR: rs = 0.443, *P* = 0.003; MLR: rs = 0.411, *P* = 0.006; SII: rs = 0.461, *P* = 0.002; SIRI: rs = 0.488, *P* = 0.010). No significant correlations were found with VDI (all *P* > 0.05). For prognosis, NLR, PLR, SII, and SIRI were positively associated with FFS 2009 (rs = 0.386, *P* = 0.011; rs = 0.366, *P* = 0.017; rs = 0.358, *P* = 0.022; rs = 0.375, *P* = 0.016, respectively), with MLR showing a trend but no significance (rs = 0.279, *P* = 0.070). Results are detailed in **Table 3**.

**Table 3:** The correlation of new inflammatory markers and different index of AAV.

Variables	NLR		PLR		MLR		SII		SIRI	
	Rs	*P*	Rs	*P*	Rs	*P*	Rs	*P*	Rs	*P*
BVAS2003	0.347	0.005	0.325	0.009	0.203	0.108	0.356	0.004	0.344	0.006
DEI	0.396	0.009	0.443	0.003	0.411	0.006	0.461	0.002	0.488	0.01
VDI	0.176	0.163	0.114	0.372	0.149	0.239	0.070	0.586	0.122	0.345
FFS2009	0.386	0.011	0.366	0.017	0.279	0.070	0.358	0.022	0.375	0.016

### Predictive capacity for active disease

**Figure 2:**
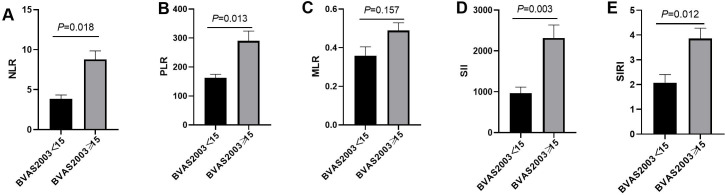
The difference of NLR, PLR, MLR, SII and SIRI in active group and non-active group. **(A)** The difference of NLR in the active and non-active group; **(B)** The difference of PLR in the active and non-active group; **(C)** The difference of MLR in the active and non-active group; **(D)** The difference of SII in the active and non-active group; **(E)** The difference of SIRI in the active and non-active group.

Patients were categorized into active (BVAS ≥ 15) and non-active groups. NLR, PLR, SII, and SIRI levels were significantly higher in the active group (*P* < 0.05), while MLR showed no difference (*P* > 0.05). **Figure 2** visually depicts these differences, showing mean levels of each marker in both groups.

**Figure 3:**
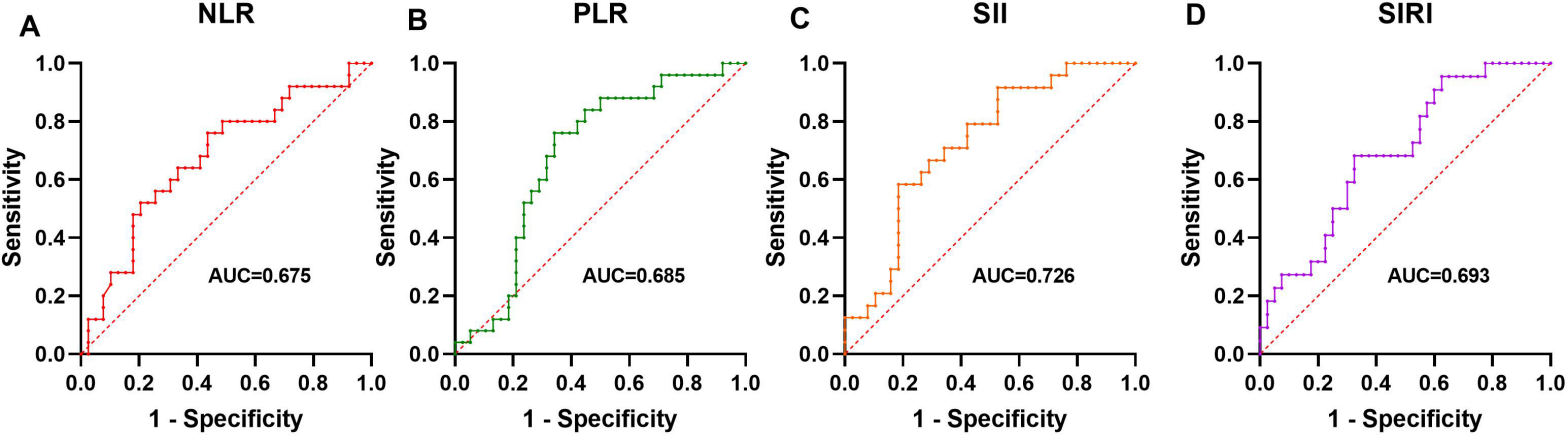
The Predictive Ability of NLR, PLR, SII, and SIRI for disease activity ROC analysis showing the ability of NLR **(A)**, PLR **(B)**, SII **(C)**, and SIRI **(D)** to predict active disease.

ROC analysis evaluated the ability of these markers to predict active disease. SII had the highest AUC (0.726, *P* = 0.003), followed by SIRI (0.693, *P* = 0.012), PLR (0.685, *P* = 0.013), and NLR (0.675, *P* = 0.019). Optimal cutoffs and performance metrics are shown in **Table 4** and visually represented in **Figure 3**.

**Table 4:** The cut-off value of NLR, PLR, SII, and SIRI for disease activity.

Variable	AUC	*P*	95%CI	Sensitivity (%)	Specificity (%)	Cut-off value
NLR	0.675	0.019	0.539-0.812	0.595	0.864	6.210
PLR	0.685	0.013	0.551-0.819	0.649	0.818	207.063
SII	0.726	0.003	0.600-0.852	0.811	0.636	822.273
SIRI	0.693	0.012	0.561-0.825	0.703	0.682	2.248

## Discussion

This study explored the utility of NLR, PLR, MLR, SII and SIRI in supporting the assessment and monitoring of AAV. Our results demonstrated that these markers effectively distinguish AAV patients from healthy controls, correlate with disease activity and disease extent, and predict active disease states.

This study demonstrates for the first time that SIRI holds significant value in differentiating AAV from healthy individuals (AUC = 0.902). In addition to SIRI, other indicators such as NLR, PLR, MLR, and SII can effectively differentiate AAV patients from healthy controls. Consistently with this study, Ahn et al. reported NLR's ability to differentiate AAV patients with high specificity (11). While these markers do not independently diagnose or classify AAV, they offer valuable complementary insights when integrated with standard clinical, histopathological, and ANCA-based criteria. Their high specificity-100% for NLR at a cutoff of 3.776 and 92.3% for SIRI at 1.059-suggests they can reinforce clinical suspicion, particularly in challenging cases like ANCA-negative AAV, which occurs in 10-20% of patients (5). Derived from routine blood counts, these markers provide a cost-effective, accessible tool to enhance diagnostic workflows, especially in resource-limited settings.

Few studies have showed that NLR, PLR can estimate the activity of AAV (11, 17, 18). To date, no studies have investigated the potential of MLR, SII and SIRI as indicators for evaluating disease activity of AAV. Our study showed NLR, PLR, SII, and SIRI correlated positively with the Birmingham Vasculitis Activity Score (BVAS), indicating their utility in reflecting AAV disease activity. The composite markers SII and SIRI, integrating multiple cell types, may capture a broader spectrum of immune dysregulation, as suggested by their strong correlations. In contrast, MLR showed no significant correlation with BVAS, suggesting that monocytes may play a limited role in driving disease activity in AAV.

The disease extent index (DEI) is utilized to assess the extent of involvement in AAV. All markers—NLR, PLR, MLR, SII, and SIRI—correlated positively with the Disease Extent Index (DEI), as reported in **Table 3**, underscoring their association with the scope of organ involvement in AAV. Multi-organ involvement, a critical determinant of disease severity, directly impacts prognosis (2). Additionally, NLR, PLR, SII, and SIRI were positively associated with the Five-Factor Score (FFS 2009), a validated prognostic tool for mortality risk (16), suggesting their potential to provide prognostic insights alongside clinical assessments. Notably, no correlations were observed with the Vasculitis Damage Index (VDI), indicating that these markers are more attuned to acute inflammation than chronic, treatment-related damage. This distinction, evident in **Table 3**, positions them as tools for monitoring active disease phases rather than long-term sequelae, a finding with significant clinical implications for acute-phase management.

Given the positive correlations between NLR, PLR, SII, SIRI, and BVAS2003, we conducted further analysis to evaluate their predictive capacity for disease activity using ROC analysis. Our results showed that SII exhibited the highest AUC (0.726), followed by SIRI (0.693), PLR (0.685), and NLR (0.675). The inclusion of platelet counts in SII may enhance its predictive power, given platelets' role in amplifying inflammation in AAV through complement system activation and interact with leukocytes and vascular endothelial cells neutrophil recruitment (19, 20). Similar predictive utility of SII has been documented in other inflammatory diseases, such as rheumatoid arthritis and systemic lupus erythematosus (21, 22). The optimal cutoffs (e.g., SII > 822.273) identified in **Table 4** offer practical thresholds for identifying active disease, potentially guiding treatment decisions. However, their moderate sensitivity and specificity suggest they should be interpreted alongside clinical assessments rather than as standalone predictors.

This study's strength lies in its comprehensive evaluation of multiple inflammatory markers, including the novel application of SIRI and SII in AAV. While NLR and PLR have been studied previously, SII, SIRI, and MLR remain underexplored, and their strong performance expands the repertoire of potential AAV biomarkers (23). The use of routine blood-derived markers, visually supported by the figures, enhances their clinical feasibility, offering a practical adjunct to existing protocols. By clarifying their supportive role, this study bridges a gap between laboratory findings and real-world applicability. However, there are some limitations in this study. This study employs a retrospective design with a relatively limited sample size. Larger-scale studies are needed to investigate the relationship between these biomarkers and clinical manifestations. As this study is retrospective in nature, it does not evaluate the potential impact of these biomarkers derived from routine blood tests on treatment outcomes. Future prospective studies are needed to investigate the predictive value of these biomarkers for treatment response.

## Conclusions

NLR, PLR, MLR, SII, and SIRI effectively distinguish AAV patients from controls and reflect disease activity, extent, and prognosis. These markers offer valuable support to standard AAV assessment, particularly in challenging cases. Integrating these markers with other biomarkers (e.g., ANCA titers, CRP) could optimize their supportive utility in AAV management. Mechanistic investigations are needed in the future to uncover specific inflammatory pathways in AAV, potentially identifying novel targets.

## Data Availability

The original contributions presented in the study are included in the article/supplementary material. Further inquiries can be directed to the corresponding author.
